# Innovative Tools for DNA Topology Probing in Human Cells Reveal a Build-Up of Positive Supercoils Following Replication Stress at Telomeres and at the FRA3B Fragile Site

**DOI:** 10.3390/cells13161361

**Published:** 2024-08-15

**Authors:** Claire Ghilain, Olivia Vidal-Cruchez, Aurélia Joly, Michelle Debatisse, Eric Gilson, Marie-Josèphe Giraud-Panis

**Affiliations:** 1 CNRS UMR7284/INSERM U1081, Institute for Research on Cancer and Aging, Nice (IRCAN), Faculty of Medicine, University Côte d’Azur, 06107 Nice, France; claireghilain@hotmail.fr; 2 Simpson Querrey Institute, 303 E Superior, Chicago, IL 60611, USA; olivia.vidalcruchez@northwestern.edu; 3 Medical Microbiology and Immunology Department, Faculty of Medicine & Dentistry, University of Alberta, 116 St. and 85 Ave., Edmonton, AB T6G 2R3, Canada; amjoly@ualberta.ca; 4 Gustave Roussy Institute, Sorbonne Université, UPMC, 94805 Villejuif, France; michelle.debatisse@gustaveroussy.fr; 5 Department of Geriatrics, Medical Center on Aging of Shanghai Ruijin Hospital, School of Medicine, Shanghai Jiaotong University, Shanghai 200025, China; 6 International Research Project in Hematology, Cancer and Aging, Pôle Sino-Français de Recherches en Sciences du Vivant et Génomique, Ruijin Hospital, Shanghai Jiao Tong University School, Shanghai 200025, China; 7 Department of Genetics, CHU, FHU OncoAge, 06000 Nice, France

**Keywords:** DNA topology, telomere, TRF2

## Abstract

Linear unconstrained DNA cannot harbor supercoils since these supercoils can diffuse and be eliminated by free rotation of the DNA strands at the end of the molecule. Mammalian telomeres, despite constituting the ends of linear chromosomes, can hold supercoils and be subjected to topological stress. While negative supercoiling was previously observed, thus proving the existence of telomeric topological constraints, positive supercoils were never probed due to the lack of an appropriate tool. Indeed, the few tools available currently could only investigate unwound (Trioxsalen) or overwound (GapR) DNA topology (variations in twist) but not the variations in writhe (supercoils and plectonemes). To address this question, we have designed innovative tools aimed at analyzing both positive and negative DNA writhe in cells. Using them, we could observe the build-up of positive supercoils following replication stress and inhibition of Topoisomerase 2 on telomeres. TRF2 depletion caused both telomere relaxation and an increase in positive supercoils while the inhibition of Histone Deacetylase I and II by TSA only caused telomere relaxation. Moving outside telomeres, we also observed a build-up of positive supercoils on the FRA3B fragile site following replication stress, suggesting a topological model of DNA fragility for this site.

## 1. Introduction

The development of high-resolution technologies to observe DNA organization, such as chromatin conformation capture or high-resolution and live microscopies, has provided evidence that DNA is constrained inside the nucleus. Topologically associated domains (TADs) where distant regions on a chromosome interact with each other to form chromatin loops, attachments of DNA to nuclear sub-structures, protein complexes and machineries, and a viscous nuclear environment limit or even block the capacity of DNA to swivel (reviewed in Racko et al. [1]). Hence, the opening of the double helix inherent to transcription and replication causes the accumulation of supercoils necessitating the enzymatic activities of Topoisomerases to relax DNA and prevent the stalling of machineries. This concerns both dividing and non-dividing cells, but proliferating cells are particularly sensitive to Topoisomerase poisons, a fact used in many chemotherapeutic cocktails. Telomeres constitute the end of linear chromosomes. In mammals, as in all vertebrates, they are composed of several kilobases of a TTAGGG motif on which a dedicated complex called Shelterin binds, harboring six proteins (TRF1, TRF2, RAP1, TIN2, TPP1, and POT1) [2]. These proteins are essential for telomere functions since they protect chromosome ends from illicit activation of the DNA damage response (TRF1, TRF2-RAP1, and POT1), protect against unwanted repair by blocking non-homologous end joining and homologous recombination (TRF2-RAP1, POT1), help replication of theses repetitive sequences (TRF1, TRF2), recruit and regulate telomerase (the enzyme that elongates telomeres in stem cells and most cancer cells (TRF1, TIN2, TPP1, and POT1)), and recruit all the proteins necessary for optimal telomeric functions such as various helicases and nucleases (TRF1, TRF2) [3]. Despite being the natural end of linear chromosomes, telomeres behave as topologically constrained structures since they are attached to the nuclear matrix [4,5], which could explain their constrained movements in the nucleus [6,7]; they are sensitive to Topoisomerase 2 inhibition [8]; they harbor structures that can constitute topological barriers blocking the diffusion and elimination of supercoils (T-loop [9,10,11] for example); they are capable of forming multicomponent liquid condensates, thus promoting phase separation [12,13]; and the telomeric repeat factor 2 (TRF2) is able to modify/recognize DNA topology through the wrapping of DNA around its central TRFH domain [8,9,14]. Moreover, in past studies, we provided direct evidence of the presence of topological constraints in telomeres through the use of a derivative of psoralen, Trioxsalen (or 4,5′,8-Trimethylpsoralen). This intercalant can crosslink DNA strands when cells are treated with UV [15,16,17]. Since it binds better on unwound DNA, it allows the detection of unwound (more relaxed, increased negative twist) regions of the genome and was used to follow genome-wide transcription in different studies [15,16]. Using this chemical, we observed telomere relaxation upon TRF2 depletion, indicating that TRF2 controls the topological constraints in telomeres [9]. This, added to the fact that TRF2 recognizes better positive supercoils than relaxed DNA or negative supercoils and that telomeres are sensitive to Topoisomerase 2 inhibition, led us to propose that telomeres are, at least transiently, under positive supercoiling constraints [8,9]. But direct proof of the presence of positive supercoils on telomeres is still lacking since Trioxsalen could only allow the monitoring of negative supercoils (relaxation and increased twist). In order to investigate both positive and negative supercoiling on telomeres in cells, we developed new and innovative probes of the topological state of DNA in cells dubbed Topotools. The analysis of their association with telomeres revealed a build-up of positive supercoiling following Topoisomerase 2 inhibition, TRF2 depletion, and replicative stress. The topological effect of replication stress was also investigated on the FRA3B fragile site where positive supercoils were also observed, giving strength to a topological model for fragility at this site.

## 2. Materials and Methods

### 2.1. Vectors

The Topotool cassette (Genscript) including the HA tag, NLS, and Topology domains was inserted into the pSFFV-GPR lentiviral vector (a kind gift of Pierre Jalinot) using the BamHI and SpeI sites. These vectors, as well as the Empty one, were used to produce lentiviruses for the transduction of HT1080 ST and HeLa-38 cells (see below).

The pSFFV-GPR vectors were used as templates in PCR reactions to allow subcloning in pET14b (for in vitro topology assays, NdeI and XbaI sites) and pSBtet-GP (sleeping beauty system Addgene #60495, SfiI sites) using the following primers:

For pET14b subcloning

GGCACCAGCGGACATATGGCCGACTACAAGGACGACGATGACAAGGGAGTG (forward primer for both Topotools)

CCAGTCAATCGCTAGCGTCGACTGTAATCCAGAGGTTGATTATCGATAAGCTTG (reverse primer for both Topotools)

For pSBtet-GP sub-cloning

GATGGCCTCTGAGGCCAACATGTTGGCCGACTACAAGGACGACGATGAC (forward primer for both Topotools)

ATATCAAGCT TGGCCTGACA GGCCACTAGT TCATCCATCG ATTGTATCCAG (reverse primer for GyrA CTD)

ATATCAAGCT TGGCCTGACA GGCCACTAGT TCATTTCTTG AATCTCCGGAC (reverse primer HMfB)

The PCR reactions were performed in conditions recommended by the manufacturer of the Taq polymerase (GoTaq^®^ DNA Polymerase, Promega).

The pSBtet-GP RFP control vector was a fortunate cloning failure of pSBtet-GP HMfB where a stop codon was inserted by the bacteria after the NLS, therefore giving an RFP protein targeted to the nucleus.

All vectors were sequenced to check for the correct sequence (Eurofins/GATC).

The in vitro topology assay was performed using supercoiled pUC19.

### 2.2. Topotool Purification

First, 1.2 L of BL21(DE3) bacteria transformed with the appropriate plasmid were grown to an absorbance of 0.8. The induction of expression was performed by adding IPTG to a final concentration of 0.1 mM and incubating it under gentle shaking for 16 h at 25 °C. Cells were then harvested and resuspended in 10 mL of the column buffer (20 mM Tris-Cl pH 8, 0.3 M NaCl, 50 mM Imidazole). After freezing the bacteria solution at −20 °C, 10 mL of the column buffer with PMSF (final concentration 1 mM) and 300 µL of an anti-protease cocktail (SIGMA P8849) were added to the frozen solution. Bacteria were sonicated at a 50% amplitude for at least 4 × 20 pulses (2 s on 2 s off) until the ice melted. The sample was then centrifugated at 38,000× *g* for 15 min at 4 °C. Next, 750 units of Benzonase (SIGMA-ALDRICH E1014) and MgCl2 with a final concentration of 1.5 mM were added to the supernatant followed by 30 min of incubation at 4 °C under gentle shaking. The solution was then loaded onto a 5 mL HisTrap^TM^ FF crude Nickel column (Cytiva) previously equilibrated in column buffer A using an FPLC Akta purifier (Cytiva). After a washing step in the same buffer, the proteins were eluted using an Imidizole gradient in the column buffer (40 min from 40 mM to 500 mM Imidazole at 1 mL/min).

Protein-containing samples were pooled and their volumes were reduced to 500 µL using a Centricon concentrator. The protein solutions were then loaded onto a Superdex 200 10/300 GL column (Cytiva). This chromatographic step was performed in 10 mM Tris-Cl pH 8 2.5 M NaCl 0.05% NP40. After several steps of concentration and dilution to decrease the amount of salt to 90 mM, the proteins were loaded onto a Mono S 5/50 GL (Cytiva). The proteins were eluted using a NaCl gradient from 100 mM to 2 M NaCl at 1 mL/min for 20 min. Pure protein-containing samples were pooled and the proteins were dialyzed against the storage buffer (50 mM Tris-Cl pH 8, 0.3 mM NaCl, 1 mM DTT, 50% glycerol). The final solution concentration was obtained by a Bradford assay, and the proteins were stored at −20 °C.

### 2.3. In Vitro Topology Assay and 2D Gel

First, 16 µg of the supercoiled pUC19 plasmid was incubated with 60 units of wheat germ Topoisomerase I (Inspiralis WGT102) in 50 mM Tris-Cl pH 8 1 mM EDTA 1 mM DTT 50 mM NaCl 20% Glycerol in a total volume of 60 µL for 90 min at 37 °C.

Then, 14 µL of this reaction was mixed with 37 µL of Reaction buffer (50 mM Tris-Cl pH 8, 100 mM NaCl, 0.1 mM EDTA, 0.8 mM DTT, 100 µg/mL acetylated BSA, and 10% glycerol) and 12 µL of either GyrA CTD (a 5 µM diluted solution in 50 mM Tris-Cl pH 8, 100 mM NaCl, 0.1 mM EDTA, 0.8 mM DTT, and 10% glycerol) or HMfB (a 9 µM diluted solution in 50 mM Tris-Cl pH 8, 100 mM NaCl, 0.1 mM EDTA, 0.8 mM DTT, and 10% glycerol) or with the buffer alone and incubated for 30 min at 37 °C. After incubation, 1 µL of 20% SDS and 1 µL of Proteinase K (Thermo Fisher Scientific 100005393) were added and incubation was continued for a further 15 min. Degraded proteins were removed by Phenol/chloroform/isoamyl alcohol extraction, and the DNA was precipitated by ethanol in the presence of 20 µg of glycogen. After recovery, the DNA was loaded onto a 1% Agarose 1 × TBE gel and migration was performed in 1 × TBE for 18 h at 3 volts/cm. After migration, the gel was soaked in a solution of 0.5 × TBE containing 1 µg/mL chloroquine. The vertical lanes containing the topoisomers were cut and placed horizontally at the top of a 15/10 cm agarose gasket, and 1% Agarose (1 × TBE 1 µg/mL chloroquine) was poured around this band. Second-dimension electrophoresis was performed at 8 volts/cm for 7 h. The gel was then stained by ethidium bromide and imaged using a Gel Doc XR+ (BioRad).

### 2.4. Cell Culture, Transfections, Lentiviral Production, and Infections

Human Embryonic Kidney 293 T, HeLa-38 [18] (a kind gift from Geneviève Almouzni), HTC116 (ATCC CCL-247 colon carcinoma cells), and super Telomerase HT1080 [19] (HT1080 ST, fibrosarcoma cell overexpressing the catalytic subunit of Telomerase, a kind gift from Joachim Lingner) cells were grown in DMEM with 10% fetal calf serum and penicillin-streptomycin at 37 °C and 5% CO_2_. VSVg-pseudotyped self-inactivating (SIN) lentiviruses, expressing an empty vector, GyrA CTD, or HMfB (pSFFV-GPR constructs), were produced by the transient transfection of a Human Embryonic Kidney 293 T cell line (by calcium phosphate precipitation) with two other packaging plasmids, p8.91 and pVSVg. Then, 36 h post-transfection, the infectious supernatant was collected, filtered, concentrated, and stored at −80 °C. The infection efficiency was investigated by determining the percentage of GFP-positive cells using FACS analysis 3 days after infection. All the infections were performed at a multiplicity of infection (m.o.i) of 2. The HCT116 Topotool-expressing cell lines were constructed using the sleeping beauty system as previously published [20] using PSBTet-GP-HMfB, PSBTet-GP-GyrA CTD, or PSBTet-GP-STOP vectors.

### 2.5. Direct Observation of Topotools in Cells

First, 0.2 million HT1080 ST cells were seeded in wells of a 6-well plate and transduced by Topotool-expressing lentiviruses 24 h after seeding. Forty-eight hours later, the cells were split into new 6-well plates containing 3 glass coverslips to a density of 0.2 million per well. They were left to recover for 24 h, and then cells were treated with RO-3306 (SIGMA SML0569) at a final concentration of 4 µg/mL for 24 h and released by changing the medium. Coverslips were recovered at different time points (between 0 and 160 min) and cells were fixed using 4% Formaldehyde in PBS (10 min of incubation at room temperature) and then rinsed 3 times with PBS. Images of the cells were acquired using a Delta vision microscope (Cytiva) by observing RFP and DAPI fluorescent signals.

### 2.6. PNA-FISH IF

Following growth and treatments on coverslips (as above), cells were fixed as above and then permeabilized by incubation for 7 min in 0.5% Triton X100 in PBS followed by 3 washes in PBS. Cells were then permeabilized by incubating for 7 min in 0.5% Triton X100 in PBS followed by three steps of dehydration using 50%, 75%, and 100% ethanol solutions in water for 3 min each. Coverslips were air dried and then 10 µL of a 0.5 µM solution of a Telo C probe coupled with the Alexa 488 fluorophore (C3TA2)3 (Panagen #F1004) in FISH buffer (70% Formamide 10 mM Tris pH 6.8 pH and 1% blocking solution–1% blocking powder Roche #11093657910 10 mM Maleic Acid 15 mM NaCl adjusted to pH 7.5) was gently deposited on the coverslips. The coverslips were laid upside down on slides and fixed with fixogum. The slides were then put on a heated plate (80 °C) for 3 min and put in a moist chamber for 4 h at 4 °C. Coverslips were recovered and washed 3 times for 7 min with the FISH buffer, 3 times for 7 min in a solution of 150 mM NaCl 50 mM Tris pH 7.5 and 0.05% Tween 20, and finally, twice in PBS before going to IF. The coverslips were put in 6-well plates, blocked with a 600 µL solution of 3% BSA, 5 % goat serum, and 0.5 % Triton X100 in PBS for 30 min at room temperature. The IF blocking solution was replaced by a fresh one and 2 µL of rat Anti-HA antibody (SIGMA #11867423001) was added before overnight incubation at 4 °C. The next day, the coverslips were washed 3 times for 7 min in PBS and incubated in 600 µL of a 450-times diluted anti-rat antibody coupled with Alexa 647 (Molecular Probes #A-21247) for 1 h at 37 °C. Final washes were performed twice using PBS. Coverslips were mounted on a slide using vectashield/DAPI, and images were acquired as above using the far-red, green, and DAPI signals. Colocalizations between the Topotool and the telomere spots were counted on slices. Only colocalization spanning at least half of the telomeric signal was counted as positive.

### 2.7. ChIP

Cells expressing Topotools (HT1080 ST or HCT116) were grown in the indicated conditions and treated with either ICRF-193 (10 µM 24 h, 35 µM 5 h) or Aphidicolin (150 nM for 24 h). Five million cells were harvested per condition and cell pellets were resuspended in 10 mL of PBS containing 1% formaldehyde and incubated for 12 min at room temperature. The reaction was stopped by adding 1.5 mL of 1 M Glycine followed by 5 min of incubation at room temperature. Cells were washed twice with PBS, resuspended in 1 mL of cell lysis buffer (5 mM PIPES pH 8, 85 mM KCl, 0.5% IGEPAL), and lysed using a dounce. After centrifugation for 5 min at 4 °C, pellets were resuspended in 500 µL of nuclei lysis buffer (50 mM Tris-Cl pH 8, 10 mM EDTA, 1% SDS) and sonicated to an average length of 500 bp using a Bioruptor (Diagenode). The sonicated samples were centrifuged at 21,000× *g* at 4 °C and transferred to 15 mL tubes. Then, 30 µL of the sample was kept aside to use as input. To the remaining solution, we added 500 µL of ChIP dilution buffer (16.7 mM Tris-Cl pH 8, 167 mM NaCl, 0.01% SDS, 1.1% Triton, 1.2 mM EDTA, anti-protease Roche #11836170001 1 tablet for 10 mL) per million cells. Next, 40 µL of protein G magnetic Dynabeads (Invitrogen #00941808) was added, and after 1 h of incubation at 4 °C on a rotating wheel, the beads were removed using a magnet. Five micrograms of rat high-affinity anti-HA (Roche #1867431) were added to each sample, and incubation was performed overnight at 4 °C. Forty microliters of beads, as above, were then added and incubated for 3 h on the rotating wheel at 4 °C. Five successive washes were performed using low-salt buffer for 10 min (150 mM NaCl, 1% triton 0.1% SDS, 2 × TE), high-salt buffer for 15 min (500 mM NaCl, 1% triton 0.1% SDS, 2 × TE), lithium wash buffer for 30 min (0.25 M LiCl, 1% NP40, 1% deoxycholic acid, 1 × TE), and then two 5 min washes in TE. The chromatin was eluted by adding 250 µL of elution buffer (0.1 M NaHCO_3_, 1% SDS), vortexed briefly, and incubated for 15 min at RT with rotation. Beads were removed using a magnet, and the elution step was repeated to obtain a final 500 µL sample in which 20 µL of 5M NaCl was added before de-crosslinking at 65 °C overnight (the input sample was treated the same way). To remove RNA, 2 µL of RNase A (10 mg/mL) was added, followed by 20 min of incubation at RT. Then, 10 µL of 0.5 M EDTA, 20 µL of Tris-Cl pH 6.5, and 2 µL of proteinase K (10 mg/mL) were added followed by incubation for 1 h at 55 °C.

DNA was recovered by phenol/chloroform extraction followed by ethanol precipitation and resuspension in 60 µL of H_2_O (100 µL for input).

### 2.8. Slot Blot

The whole 60 µL of ChIP samples above and the input samples (5 µL of input and 55 µL of elution buffer) were diluted with 2 volumes of 1.5 M NaOH and 1 M NaCl followed by 10 min of incubation at RT, and then diluted 10 times in 0.1 × SSC, 0.125 M NaOH and incubated on ice for 5 min.

The samples were then loaded onto a Hybond N+ membrane using a slot blotter. The membrane was neutralized by adding 300 µL of 0.5 M Tris-Cl pH 7.5, 0.5 M NaCl.

After crosslinking the membrane (0.12 J/cm^2^), the membrane was humidified for 10 min in 0.25 M Na Phosphate buffer pH 7.2 at 50 °C. Prehybridization was performed in 6 × SSC, 0.5% SDS, and 1% dried skimmed milk. A ^32^P-labeled telomeric probe was added and left to hybridize overnight at 50 °C. After 4 washes at 50 °C (2 × SSC 10 min, 2 × SSC 1% SDS 30 min, twice 0.2 × SSC 1% SDS 30 min), the membrane was dried and exposed to a phosphorimager screen (Cytiva), and the signal was detected using a Typhoon FLA 9000 (Cytiva). Quantifications were performed using the Image Quant software (Cytiva).

### 2.9. qPCR

qPCR was used to quantify *FHIT* DNA in ChIP samples using A StepOne apparatus from Applied Biosystems. QPCR reactions were performed as recommended by the manufacturer of the Master Mix (Roche #4913914001) using a final concentration of 0.3 µM of the following forward (for) and reverse (rev) primers:
A forCAGCCACCCTTCCTTACTGGA revGCCACTAGAGTCAGCCAAGGB forCCTCTTTGCCACACACTTGCB revTAATTGGCACAGGGCCTGACC forGAGTCAACAGAGTGGACCTACCC revTAGATGACCGGAAGGTGTGTTD forCCAGCAGTTAATGGCTTGCTD revGTTGGGCCATGACCAGTTACE forGTAAACAATGCAGGATCACCGTGTAE revTTCCACCTACTTTGGGCCTGAGF forTGTGGTCATCACCAACCCAGF revCAGGTTAGCAGGTCTCGGTGSub-telo forCCCAAACCCTAACCCTAAAASub-telo revCTTCCTGTTTGCAGCACTGA

Primers were designed using the Primer 3 BLAST software (https://www.ncbi.nlm.nih.gov/tools/primer-blast/).

### 2.10. Immunoblots

Cells were lysed using a RIPA solution for 30 min at 4 °C. After centrifugation, the supernatant was collected, and the number of proteins was quantified by the method of Micro-Bradford using BSA as the control protein. The Laemmli buffer was added, and proteins were boiled for 5 min at 90 °C. Then, 50 µg of the protein sample was were separated by SDS-PAGE and transferred to and probed with anti-tRFP antibody (rabbit AbCam ab233, 1/500 dilution), anti-Beta-Actin (mouse SIGMA A5441, 1/500 dilution), or anti-Tubulin (mouse SIGMA T51681/500 dilution) followed by secondary detection with dye-conjugated antibodies (anti-rabbit IRdye 680 Li-Cor 926-32221, anti-mouse IRdye 800 Li-Cor 926-32210, diluted at 1/20,000) to be detected by Odyssey.

### 2.11. qRT–PCR for Measuring TRF2 Expression

Total RNA was extracted with the RNeasy miniprep kit (Qiagen) using on-column DNase digestion (RNase-Free DNase Set, Qiagen). The RNA concentration was determined by measuring the OD at 260 nm. For quantitative RT–PCR (qRT–PCR), cDNAs were prepared using the High-Capacity RNA-to-cDNA Master Mix (Applied Biosystems) followed by Stepone qPCR, using the Fast SYBR^®^ Green Master Mix (Applied Biosystems), performed as per the manufacturer’s instructions. As a control for DNA contaminations, RT–PCR without RT was performed in parallel for each sample, which did not result in detectable PCR products.

TERF2 Forward primer GCCCATCCTGTTATCCAGAA

TERF2 Reverse primer CAAAGCCTTTTTGGCCATC

The housekeeping gene used as a control was RPLP0 (Ribosomal Protein Lateral Stalk Subunit P0) and the primers used were:

RPLP0 forward AACTCTGCATTCTCGCTTCCT

RPLP0 reverse ACTCGTTTGTACCCGTTGATG

Quantifications were performed using the Pfaffl equation.

### 2.12. siRNA KD of TERF2 mRNA

siRNA (On-Target Plus SMARTpool, Dharmacon L-003546-00-0005) transfections were performed with the Dharmafect1 transfection reagent (Dharmacon) for 72 h according to the manufacturer’s instructions.

## 3. Results

### 3.1. GyrA CTD and HMfB-Based Topotools to Investigate DNA Topology Inside the Nucleus

In order to investigate DNA topology in human cells, we took advantage of the intrinsic properties of two prokaryotic proteins to recognize either positive or negative supercoiling in DNA. The C-terminal domain of Gyrase A (GyrA CTD) from *Escherichia coli* is essential for the activity of this topoisomerase since it allows the recognition and binding of Gyrase on its supercoiled template and the correct entry of DNA in the DNA gates of the enzyme. This is achieved through a one-turn wrapping of DNA around the domain in a right-handed orientation [21]. Conversely, HMfB, a protein from the archae *Methanothermus fervidus*, also recognizes negative supercoiling [22] through one-turn DNA wrapping but in the opposite direction (i.e., left-handed wrapping). In this archae, HMfB plays the structural role of nucleosomes, hence its designation as a Histone-like protein [23]. A BLAST search did not reveal any orthologs of these domains in eukaryotes.

**Figure 1:**
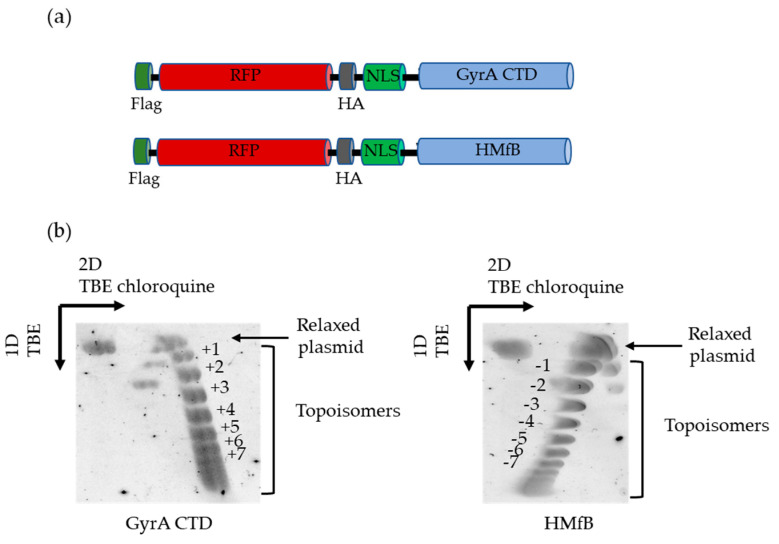
In vitro topological properties of Topotools. (**a**) Schematics of Topotools proteins. For purification purposes, recombinant Topotools were fused to an N-terminal (His)6 tag. (**b**) 2D Gels of Topo assays where 1 µM Topotools were incubated with a relaxed circular plasmid in the presence of wheat germ Topoisomerase I. Note the appearance of topoisomers and their inverse orientation. Chloroquine, which creates (+) supercoils, increases migration for RFP-GyrA CTD topoisomers, indicating that RFP-GyrA CTD also creates (+) supercoils. In contrast, chloroquine slows down migration for RFP-HMfB topoisomers, indicating that RFP-HMfB creates (−) supercoils.

To detect these prokaryotic protein domains in cell nuclei and protein extracts, we added to the sequence an array of three nuclear localization signals (NLS), both a Flag and HA tag, and we fused the proteins to RFP, producing DNA topology-sensing proteins that we called Topotools (Figure 1a). As the first control, we verified that the addition of all these elements did not modify GyrA CTD and HMfB intrinsic properties to modify DNA topology in the presence of wheat germ Topoisomerase I, a consequence of DNA wrapping. An N-terminal Histidine tag was added to the protein sequence to allow Affinity chromatography purification (Figure S1), and topology assays were performed using 1 µM of each recombinant Topotool. As seen in Figure 1b, the incubation of a relaxed circular plasmid with the Topotools (top left of the 2D gels) gave rise to several topoisomers visible as bands migrating at different speeds in the TBE buffer (first/vertical dimension). Each topoisomer bears a finite number of superhelical turns, starting with one turn for the band just below the relaxed plasmid. Thus, in our conditions, Flag-RFP-HA-GyrA CTD and Flag-RFP–HA-HMfB (called GyrA CTD and HMfB henceforth) were able to create up to about ten superhelical turns. The second, horizontal dimension performed in TBE in the presence of chloroquine allowed the determination of the chirality of these turns (right + or left −). Indeed, this intercalant created positive supercoils and therefore increased the migration of positive topoisomers, producing an arc to the right from the relaxed plasmid at the top (GyrA CTD), and decreased the migration of negative topoisomers, producing an arc to the left (HMfB). As expected, the GyrA CTD Topotool creates topoisomers with positive supercoils while the HMfB Topotool creates topoisomers with negative supercoils and thus are wrapped by DNA in a right-handed or left-handed orientation, respectively. This will allow them to recognize either positive/right writhe (GyrA CTD) or negative/left writhe (HMfB) as they do in their native contexts.

### 3.2. Topotools Are Nuclear and Bind Chromatin

To study Topotool binding on DNA in human cell nuclei, we constructed two types of vectors. A lentiviral construct (Figure S2) where Topotool mRNAs are expressed under the control of an SFFV promoter as a cistronic module also containing sequences coding for GFP and Puromycin acetyltransferase. These sequences are separated by the viral E2A and T2A peptides sequences, allowing ribosomal skipping during translation and thus coordinated but separated expression of GFP, Puromycin acetyltransferase, and Topotools. Since neither GFP nor Puromycin acetyltransferase bears an NLS, only Topotools are nuclear. Hence, GFP in all constructs and RFP in the empty-vector context remain in the cytoplasm (Figure S2c). These constructs were used to transduce HeLa-38 and HT1080 ST cells (see Section 2). The second type of cell system is based on an inducible system, transduced in HCT116 cells, and using a Doxycycline (DOX)-inducible Tet promoter for Topotool expression (PSBtet-GP [20], Figure S3). In this cell system, we also expressed a truncated version of Topotools missing the GyrA CTD or HMfB domains and called it RFP (Figure S3b). The inducible Topotool cassettes were randomly integrated into the genome of HCT116 (see Section 2). The addition of Doxycycline triggered the expression of the Topotools and RFP and their localization in the nuclei (Figure S3c).

**Figure 2:**
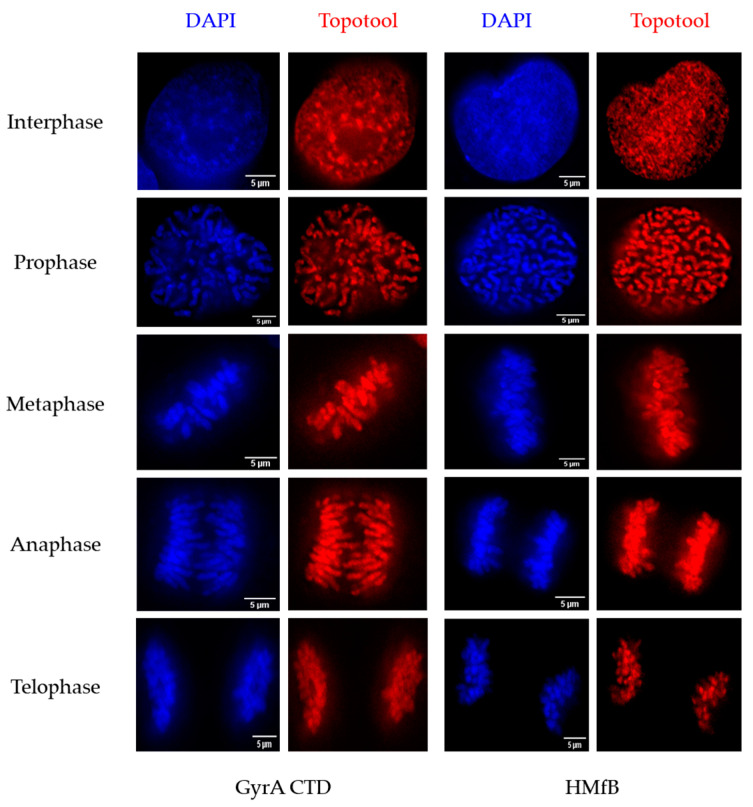
Topotools bind chromatin all along mitosis. Representative images of fixed Topotools-expressing HT1080 ST cells synchronized by treatment with RO-3306 and then released. Images were taken at different time points, allowing observation of different stages of mitosis. Cells were fixed and no labeling or IF were performed. DAPI and Topotools RFP signals were monitored.

To test if the Topotools could bind chromatin, we looked at mitotic cells in the different cell lines (Figure 2 and Figure S4). Direct (without immuno-detection) fluorescence analyses using confocal microscopy revealed perfect co-localization between DAPI and Topotools, but not RFP, at all stages of mitosis, indicating chromatin binding.

This binding also indicated that mitotic chromosomes contain both right-handed and left-handed loops. Of note, we observed rather intense staining at some of the extremities of the chromosomes, suggesting telomeric binding. Such telomere binding was confirmed by the examination of Topotools localization on metaphase spreads by PNA-FISH IF (Figure S5).

### 3.3. Topoisomerase 2 Inhibition Causes a Build-Up of Positive Supercoils at Telomeres

**Figure 3:**
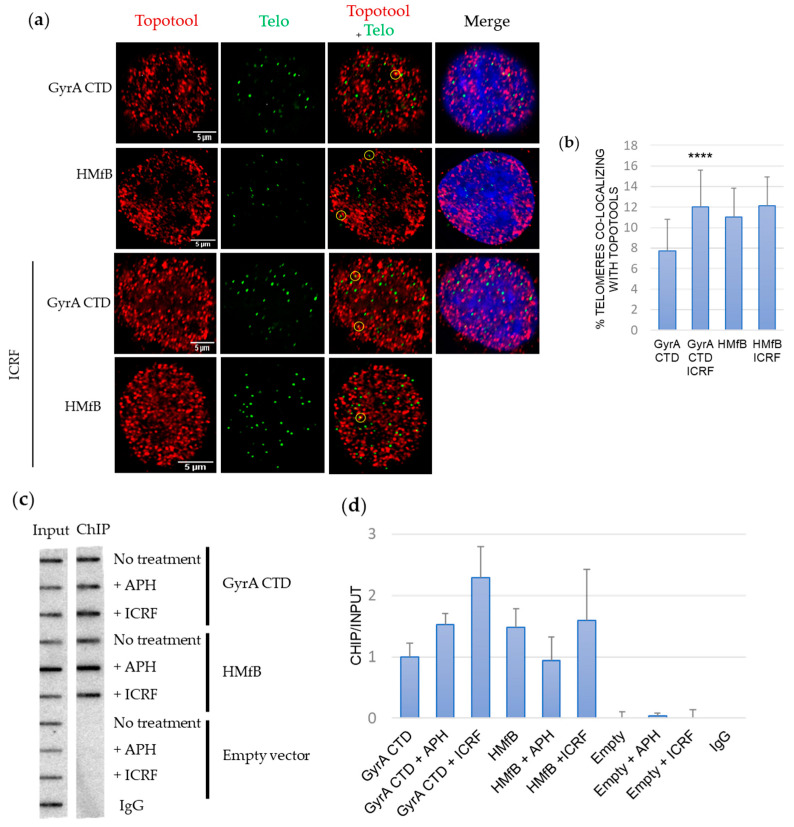
Topoisomerase 2 inhibition and replication stress cause a build-up of positive supercoils on telomeres. (**a**) Representative images of Hela-38 cells transduced by Topotools-expressing lentiviruses and treated with ICRF-193 at 35 µM for 5 h. Topotools were detected by IF using a rat anti-HA antibody and telomeres by PNA FISH using a telomeric probe. Circles indicate co-localizations. (**b**) Quantification of the co-localizations between Topotools and telomeres observed in the experiment shown in (**a**). Statistics were performed compared to control condition using Kruskal–Wallis followed by Dunn tests. **** *p* < 0.0001. (**c**) Representative Slot blot of ChIP experiments performed with HT1080 ST transduced by Topotools-expressing or Empty lentiviruses. Cells were treated using 150 nM of Aphidicolin and 10 µM of ICRF-193 for 24 h. Topotools-bound chromatin was immuno-precipitated using an anti-HA antibody. (**d**) Quantification of the ChIP experiment. Error bars represent maximal values obtained between two biological replicates.

Topoisomerase 2 comes in two flavors in vertebrate cells: Topoisomerase 2 alpha and 2 beta. Topoisomerase 2 alpha is mainly expressed during the S phase and is an essential protein in proliferating cells. Topoisomerase 2 beta mainly acts during transcription and is dispensable in mammalian cells (for a review, see Pommier et al. [24]). From a biochemical point of view, they are both “writhases” (decatenating DNA) and act at the cross-over between double strands. Topoisomerase 2 alpha is specialized in removing positive supercoils ahead of replication forks, as well as decatenating newly synthesized strands behind. Topoisomerase 2 beta also decatenates DNA but has no preference between positive and negative topology. Their inhibition causes chromatid entanglements and the accumulation of unresolved knots. Thus, inhibiting Topoisomerase 2 alpha and 2 beta constitutes an ideal way to test the capacity of GyrA CTD to sense positive topological stress. For this inhibition, we treated the cells with ICRF-193, a catalytic inhibitor of both topoisomerases [24], at a low concentration to avoid cell death without avoiding DNA damage (10 or 35 µM). We checked for Topotools binding by analyzing co-localizations between telomeres (detected by PNA-FISH) and Topotools (by IF using a Rat anti-HA antibody) using confocal microscopy on Topotools-expressing HeLa-38 cells (Figure 3a,b and Figure S6a) and ChIP-slot blot on Topotools-expressing HT1080 ST cells (Figure 3c,d, Figures S6b and S7).

Both experiments showed an increase in GyrA CTD binding in these stress conditions while the binding of HMfB was not affected, indicating a build-up of positive topology. These experiments show that telomeres can harbor positive supercoils. They also validate GyrA CTD as a valuable tool for the analysis of positive supercoils.

### 3.4. Replicative Stress Causes a Build-Up of Positive Supercoils at Telomeres

Replication fork stalling is a major issue in regions of the genome that are hard to replicate such as repetitive and/or G-rich sequences (e.g., telomeres and pericentromeric heterochromatin [25,26,27]). It is generally admitted that a build-up of supercoils ahead of the fork caused by the presence of topological barriers or overloading/inhibition of topoisomerases (see above) will slow down replication. Here, we wondered if the reverse could also be true, i.e., whether fork stalling can cause topological changes in the DNA. Therefore, we treated Topotools-expressing HeLa-38 and HT1080 ST cells with Aphidicolin (APH, a specific inhibitor of DNA polymerase alpha and delta) and tested Topotools binding on telomeres using ChIP, as above. Figure 3c and Figure S7 show that APH increases GyrA CTD binding. HMfB recruitment, on the other hand, tends to decrease.

### 3.5. Trichostatin-A Treatment Causes an Increase in HMfB Binding

**Figure 4:**
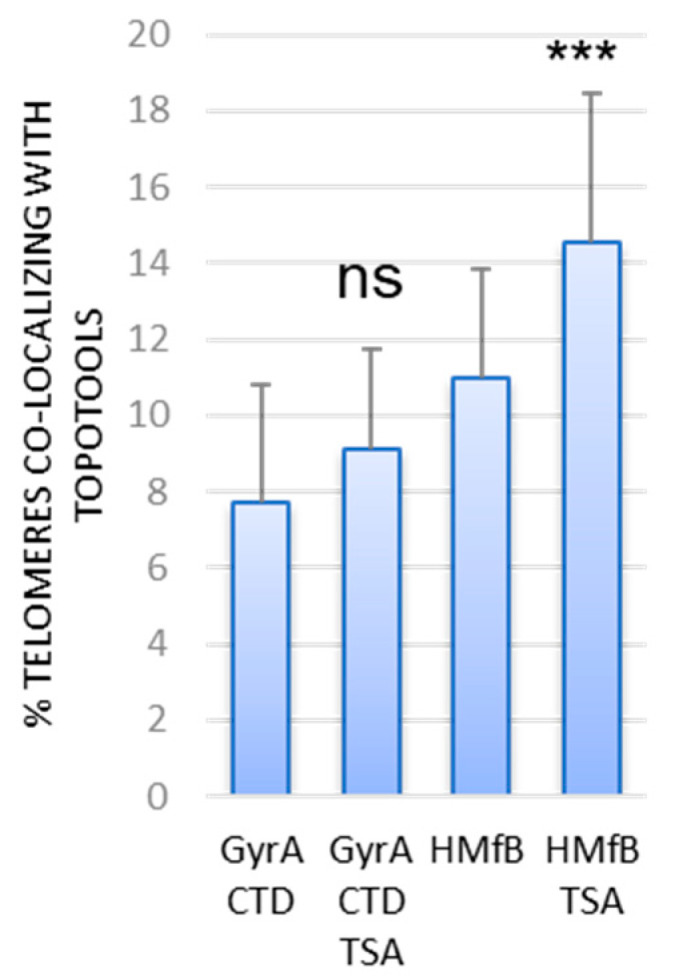
Inhibition of HDAC I and II by Trichostatin-A (TSA) causes telomere relaxation. Quantification of the co-localizations between Topotools and telomeres observed in Hela-38 cells transduced by Topotools-expressing lentiviruses and treated with 2.5 µM of TSA for 5 h. Topotools were detected by IF using a rat anti-HA antibody and telomeres by PNA FISH using a telomeric probe. Statistics were performed compared to control condition using Kruskal–Wallis tests followed by Dunn tests. *** *p* < 0.001.

Trichostatin-A (TSA) is an inhibitor of Histone deacetylases I and II and thus causes hyperacetylation of histones. It alleviates the repression of genes or transgenes located in the sub-telomeric region of cancer cells [28,29,30]. Perhaps connected to the former, it was also shown to cause a loss of T-loops in HeLa cells [31]. Both hyperacetylation of histones and T-loops loss are expected to decrease telomere compaction, an effect potentially detectable through HMfB binding. Accordingly, we observed a small but significant increase in the binding of this Topotool when treating HeLa-38 cells with TSA (Figure 4 and Figure S8).

### 3.6. TRF2 Depletion Has a Dual Effect on Telomere Topology

**Figure 5:**
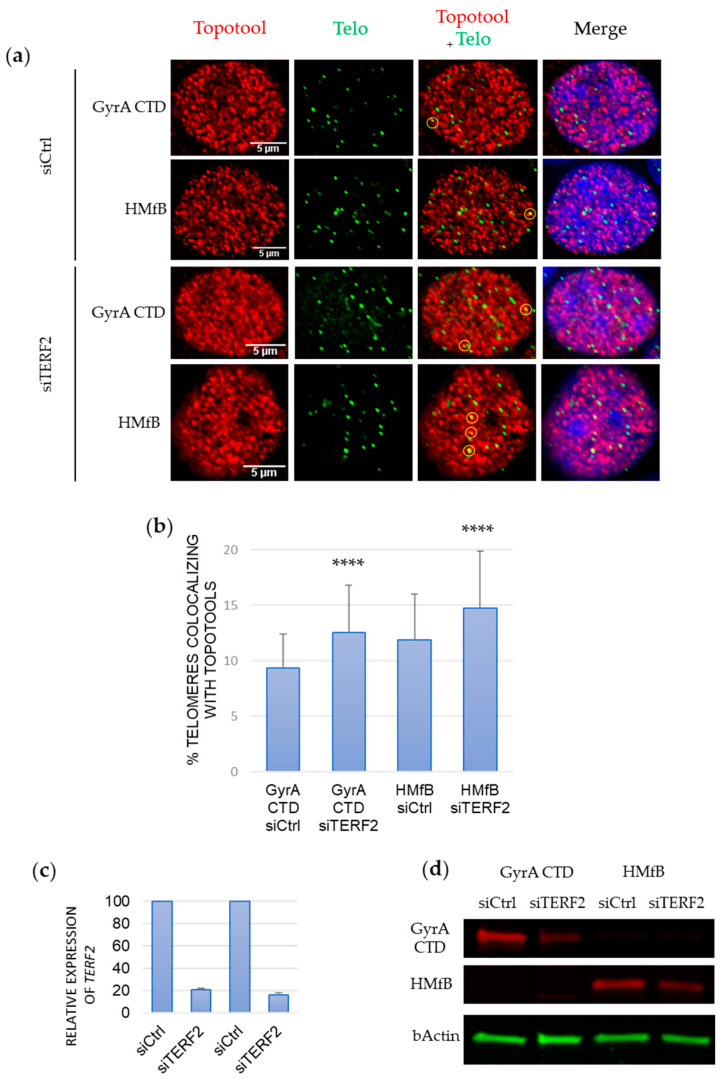
Knockdown of TRF2 causes supercoil build-up on telomeres. (**a**) Representative images of HCT116 cells expressing the corresponding Topotools and transfected with vectors expressing either a siRNA targeting *TERF2* mRNA or a siRNA of identical composition but with a random sequence (siCtrl). Topotools were detected by IF using a rat anti-HA antibody and telomeres by PNA FISH using a telomeric probe (Telo). Circles indicate co-localizations. (**b**) Quantification of the co-localizations between Topotools and telomeres observed in the experiment shown in (**a**). Statistics were performed compared to control conditions using Kruskal–Wallis tests followed by Dunn tests. **** *p* < 0.0001. (**c**) QPCR analysis of TRF2 expression in the HCT116 cells used in (**a**). (**d**) Western blot showing Topotools and Actin expression in the experiment shown in Figure (**a**). Anti-tRFP and anti-Actin antibodies were used.

Through analysis of the role of TRF2 in replication on telomeres and pericentromeric sequences, we previously found that TRF2 assists Topoisomerase 2 [8] and serves as an accessory protein for replicating repetitive, G-rich sequences of pericentromeric heterochromatin [26]. Based on TRF2’s capacity to recognize positively supercoiled DNA and the replicative stress observed in its absence, we proposed that TRF2 is recruited in the case of positive supercoil build-up caused by Topoisomerase 2 overload or replication blockage [8,25,26]. In this model, once bound, TRF2 would recruit activities necessary to overcome the problem, such as the nuclease Apollo [8]. Thus, an increase in positive supercoils should be detected in the absence of TRF2. However, previous Trioxsalen experiments indicated that TRF2 depletion causes telomere relaxation/decompaction (an increase in Trioxsalen binding) [9]. This suggests a possible dual function of TRF2 regarding telomere topology. To investigate this idea, we analyzed the binding of our Topotools on telomeres following the knockdown of TRF2 expression by siRNA silencing of its gene *TERF2*. As seen in Figure 5 (and Figure S9 for a biological replicate using a different antibody), the recruitment of both Topotools is enhanced upon *TERF2* expression knockdown, indicating both an increase in negative supercoils and a build-up of positive topology on telomeres, thus confirming the dual functions of TRF2.

### 3.7. Replicative Stress Causes Topological Changes in the Fragile FRA3B Site

**Figure 6:**
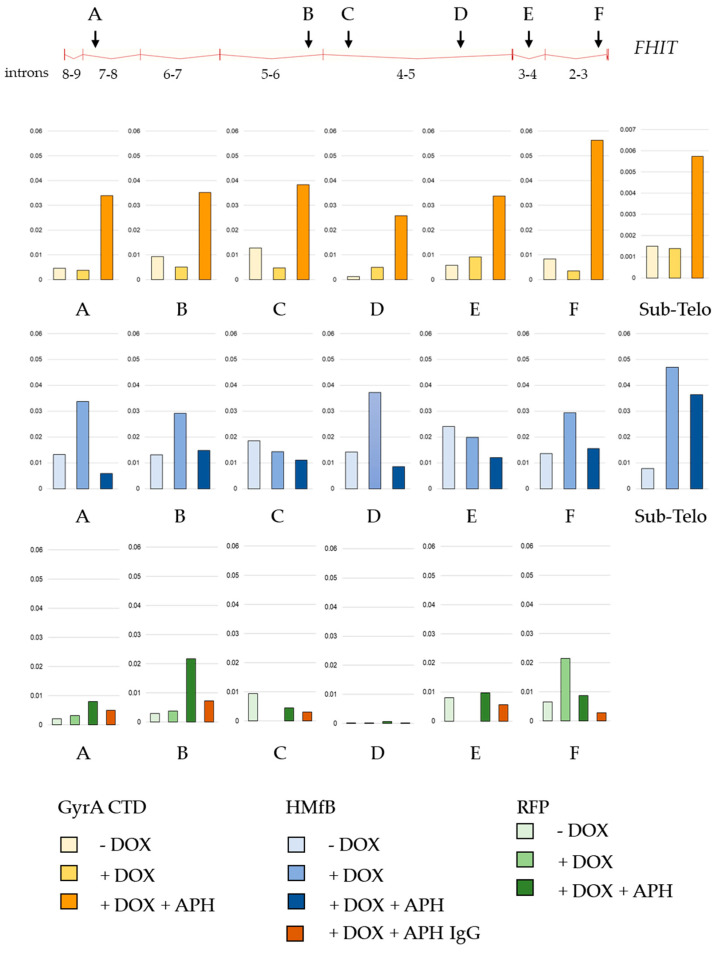
Mild replication stress causes topological changes in the *FHIT* gene. Top: graphical representation of the *FHIT* gene and position of primers pairs used to analyze ChIP samples. Bottom: quantitative analysis of ChIP samples by qPCR using primers pairs A to F and performed on HCT116 cells expressing the Topotools and the RFP control. Three conditions were analyzed: DMSO (− DOX), Doxycyclin induction of Topotool expression (+ DOX), and Doxycyclin induction and treatment with Aphidicolin (150 nM for 24 h, + DOX + APH). Antibodies used were rat anti-HA and rat IgG.

Common fragile sites are large genomic regions particularly sensitive to replicative stress and prone to breakage due to the failure to completely replicate before mitotic entry [32]. This fragility is now perceived as being the consequence of the combined effects of a paucity of origins in these regions increasing both the risk of forks slowing and the difficulty of rescue [33], the presence of a TAD boundary in large and transcribed genes, and a delay in replication timing [34]. Since TAD boundaries are suggested to accumulate torsional stress [35,36] and since, as shown above, replication stress by treatment with APH causes a build-up of positive supercoiling on telomeres, we wondered if changes in the DNA topology could also be part of the fragility process. Therefore, we treated HCT116 cells expressing Topotools through Doxycycline induction (DOX) with a low concentration of APH (150 nM) and analyzed Topotool binding by ChIP-qPCR on FRA3B [33]. FRA3B is a fragile site known to be sensitive to a low dosage of APH in HCT116 cells and contained in the extremely long *FHIT* gene (1.5 Mb) [37] on chromosome 3. Figure 6 and Figure S10 (for a biological replicate of the experiment shown in Figure 6) show the qPCR analysis of ChIP experiments using different pairs of intronic primers. We observed little difference in the amount of DNA recovered with or without DOX for GyrA CTD, indicating limited binding of this Topotool in normal conditions. For HMfB, on the other hand, immunoprecipitated DNA is increased following induction, indicating some degree of binding and, thus, the presence of negative supercoils likely due to the transcription of this gene. Treating cells with APH results in an important increase in GyrA CTD while the binding of HMfB is decreased, pointing to a net increase in positive supercoils.

## 4. Discussion

For a long time, since the “twin-domain” model of Liu and Wang introduced the idea of opposite supercoils created ahead and behind an advancing RNA polymerase [38,39], DNA topology was considered a matter concerning only circular genomes. In eukaryotes, it was thought to be transient, efficiently dealt with by topoisomerases, or stored by nucleosomes. Nowadays, with the discovery of TADs, the characterization of proteins able to detect overwound or underwound DNA besides Topoisomerases (with TRF2 being one of those) and the growing number of topological barriers blocking the diffusion of topological constraints (TADs boundaries, machineries, attachments to nuclear sub-structures, R-loops, T-loops, etc.), the importance of DNA topology in the functioning of eukaryotic genomes has become a topic that rose from the shadows. This began with the design of tools and methodologies to tackle the question. More than 10 years ago, Trioxsalen, which binds and crosslinks unwound regions of the genome, allowed the analysis of changes in DNA topology occurring during transcription, i.e., negative supercoiling in the form of variations in twist [15,16]. But this only gave a partial image of DNA topology since neither negative writhe nor positive supercoiling (twist and writhe) could be investigated with this chemical. The Topotools described here provide such missing information. Based on prokaryotic proteins that recognize writhe, both negative (HMfB) and positive (GyrA CTD), they allow the monitoring of supercoils in human nuclei since they are readily visualized by microscopy (by IF or directly thanks to their RFP moiety) and immunoprecipitated and immunodetected in cells and Western blots. As the first approach, we decided to study telomere topology since we had already analyzed Trioxsalen binding on these regions and revealed the relaxation effect of TRF2 depletion [9]. In vitro characterization of the Topotools showed expected behaviors, i.e., GyrA CTD creates positive supercoils while HMfB does the opposite, indicating that the addition of tags and RFP did not modify the intrinsic properties of these proteins (Figure 1). When expressed in human cancer cell lines, they are both nuclear and bind chromatin all along mitosis, suggesting the presence of both topological states in mitotic chromosomes (Figure 2). In the interphase, they present a punctuated pattern by IF (Figure 3 and Figure 5), allowing us to analyze their binding on telomeres both by monitoring their co-localization with telomeres using confocal microscopy and by ChIP. Treating cells with ICRF-193 allowed us to validate GyrA CTD. Indeed, as ICRF-193 is an inhibitor of Topoisomerase 2, we expected an increase in positive supercoils, which is exactly what we observed through the increase in GyrA CTD binding (Figure 3). We also analyzed Topotool binding during replication stress and observed an increase in GyrA CTD recruitment, showing a build-up of positive topology when slowing forks with Aphidicolin. Relieving topological stress during replication essentially relies on two mechanisms: the action of topoisomerases and fork rotation, with the latter allowing the diffusion of supercoils from the front (un-replicated DNA) to the rear (newly replicated DNA) of the fork, thus creating pre-catenanes (entangled, newly replicated strands). This rotation was shown to be finely regulated in yeast cells and to occur mainly during termination and during elongation in hard-to-replicate and fragile loci [40]. Although telomeres were not studied in this report, human telomeres definitively fit this model. It is therefore possible that fork rotation occurs more readily on telomeres and, thus, slowing the fork would inhibit rotation and cause a build-up of positive supercoils ahead of the fork. It would be interesting to test this hypothesis by looking at replication intermediates in the presence of these drugs, as was performed many years ago by Nicholas Cozarelli [41]. Another region of the human genome that clearly fits this model of fragility is the bona fide common fragile site FRA3B. Our experiments show that Aphidicolin also causes a build-up of positive topology at this site. It is therefore tempting to add the increase in positive topology as a signature of fragility. In accordance, the inhibition of Topoisomerase 2 was shown to decrease APH-dependent breakages in FRA3B and the RET oncogene [42]. It may also be hypothesized that variations in topology may be part of the damage process for other drugs such as methyl-methane sulfonate, cis-platinum, etoposite, hydroxyurea, and camptothecin, for example. An analysis of Topotool binding in these contexts would also be very informative.

As mentioned above, TRF2 depletion causes telomere relaxation, indicated by an increase in HMfB binding, but we also observed an increase in GyrA CTD binding, thus revealing a build-up of the opposite topology. This duality might seem surprising, but telomeres span several kilobases and may contain several domains where TRF2 depletion can have different topological effects: the terminal part of telomeres holds the T-loop, the lasso-like structure of which the formation is dependent on TRF2. Removing TRF2 would cause the opening of the loop and, thus, relaxation of this part of the telomere (increase in HMfB binding). In internal parts of telomeres, TRF2 depletion could have the opposite effect by making the removal of positive supercoils more difficult (increase in GyrA CTD binding).

A recent article [43] reported the analysis of positive supercoiling in *Escherichia coli* and *Saccharomyces cerevisiae* using GapR, a chromosome-structuring protein from *Caulobacter crescentus* [44]. These authors found GapR binding in yeast centromeres, cohesin binding sites (likely linked to TAD boundaries), ARS (Autonomously Replicating Sequences, likely compensating for the extreme underwinding of replication origins), and amazingly, an enrichment of yeast telomeres described as higher than other transcribed regions. Our results indicate that human telomeres can also harbor positive supercoils showing that telomeres are topologically constrained in species as evolutionary, apart from in yeast and humans. A comparison between GyrA CTD and GapR would thus be very informative. Indeed, GyrA CTD and GapR are expected to be complementary rather than redundant. GapR recognizes over-twisted DNA (~8.5°) [44] while GyrA CTD recognizes positive writhing and, thus, can detect plectonemes and catenanes undetectable by GapR. Hence, the use of both tools would provide a complete picture of positive topology and, combined with HMfB (which detects negative writhing) and Trioxsalen (which detects under-twisting), would provide the ultimate picture of DNA topology in cells.

## 5. Conclusions

In conclusion, we have constructed and validated new and innovative tools, named Topotools, to study DNA topology inside cells. These tools constitute an invaluable addition to the existing tools Trioxsalen and GapR that monitor un-winding or over-winding, respectively, since, in contrast to the formers, they provide an image of the negative and positive writhe (catenanes and plectonemes) of the studied genome. Thanks to these tools, we were able to analyze the topology of telomeres and the FRA3B fragile site in human cancer cells and reveal changes in topology caused by replicative stress and topoisomerase inhibition.

## Data Availability

No new data were created.

## Supplementary Materials

The following supporting information can be downloaded at: https://www.mdpi.com/article/10.3390/cells13161361/s1. Figure S1: Recombinant Topotools. Figure S2: Constructs and expression of Topotools using lentiviruses. Figure S3: HCT116 Doxycyclin-inducible system. Figure S4: Topotools and chromatin during mitosis. Figure S5: Partial telomeric localization of Topotools on metaphasic chromosomes. Figure S6: ICRF and APH do not cause alterations in Topotool expression. Figure S7: Topoisomerase 2 inhibition and replication stress cause a build-up of positive supercoils on telomeres. Figure S8: Trichostatin A (TSA) does not cause alterations in Topotool expression. Figure S9: Knockdown of TRF2 causes supercoils build-up on telomeres. Figure S10: Mild replication stress causes topological changes in the FHIT gene.

## Abbreviations

T-loop	Telomeric loop a lasso-like structure ending telomeres of most species
R-loop	RNA loop, a loop created by the retention of an RNA transcript on a transcribed region
HA tag	tag derived from the human influenza hemagglutinin (HA) protein
NLS	Nuclear Localization Signal
RFP	Red Fluorescent Protein
IPTG	Isopropyl β- d-1-thiogalactopyranoside
PMSF	phenylmethylsulfonyl fluoride
PNA-FISH	Peptide nucleic Acid—Fluorescence in situ Hybridization
IF	Immuno-Fluorescence assay
ChIP	Chromatin Immuno-Precipitation
qPCR	quantitative Polymerase Chain Reaction
qRT-PCR	Reverse transcription followed by qPCR
KD	knock-down
