# Supplementary material for: Innovative Tools for DNA Topology Probing in Human Cells Reveal a Build-Up of Positive Supercoils Following Replication Stress at Telomeres and at the FRA3B Fragile Site
Source: Cells. 2024 Aug 15;13(16):1361. doi: 10.3390/cells13161361 (PMC11352870; doi:10.3390/cells13161361)
Supplement: Supplementary file 1 [file cells-13-01361-s001.zip › cells-3148266-supplementary.pdf]

Supplementary Figures

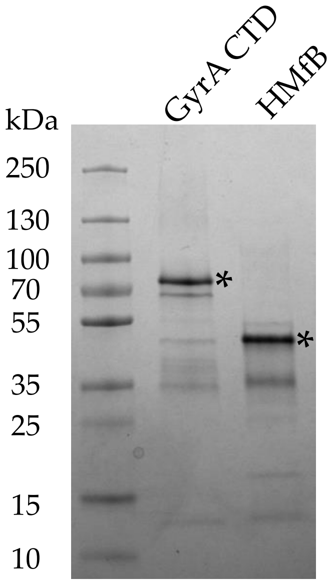

Figure S1. Recombinant Topotools. Coomassie stained SDS PAGE of purified recombinant Topotools. Full length proteins are indicated by asterisks.

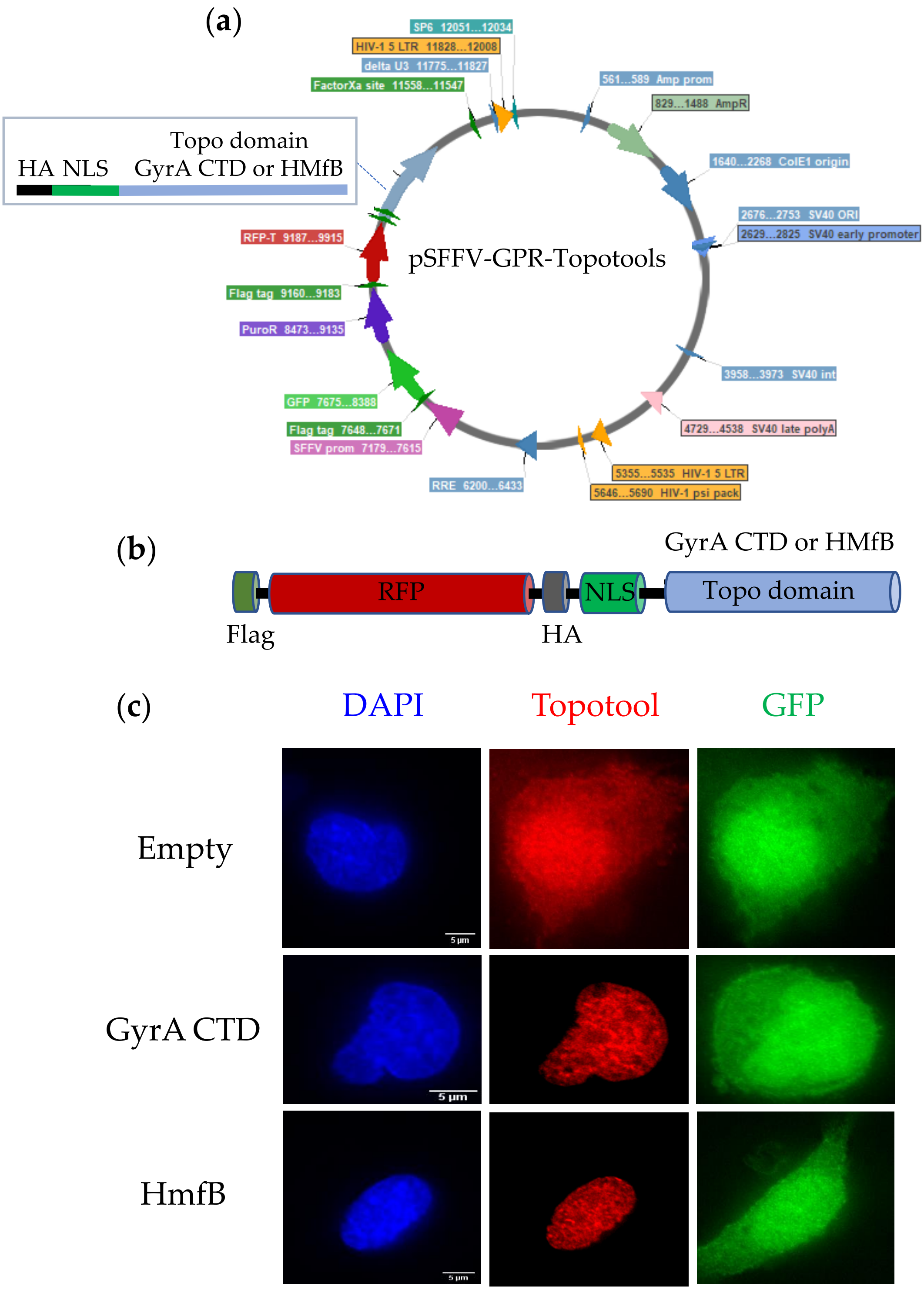

Figure S2. Constructs and expression of Topotools using lentiviruses. (**a**) Lentiviral constructs used to transduce HT1080St and HeLa-38 cells. (**b**) Schematics of the Topotools produced by this system. (**c**) Representative confocal images of fixed HT1080 ST cells transduced by Topotools (GyrA CTD or HMfB) expressing or Empty lentiviruses. No labelling or IF were performed.

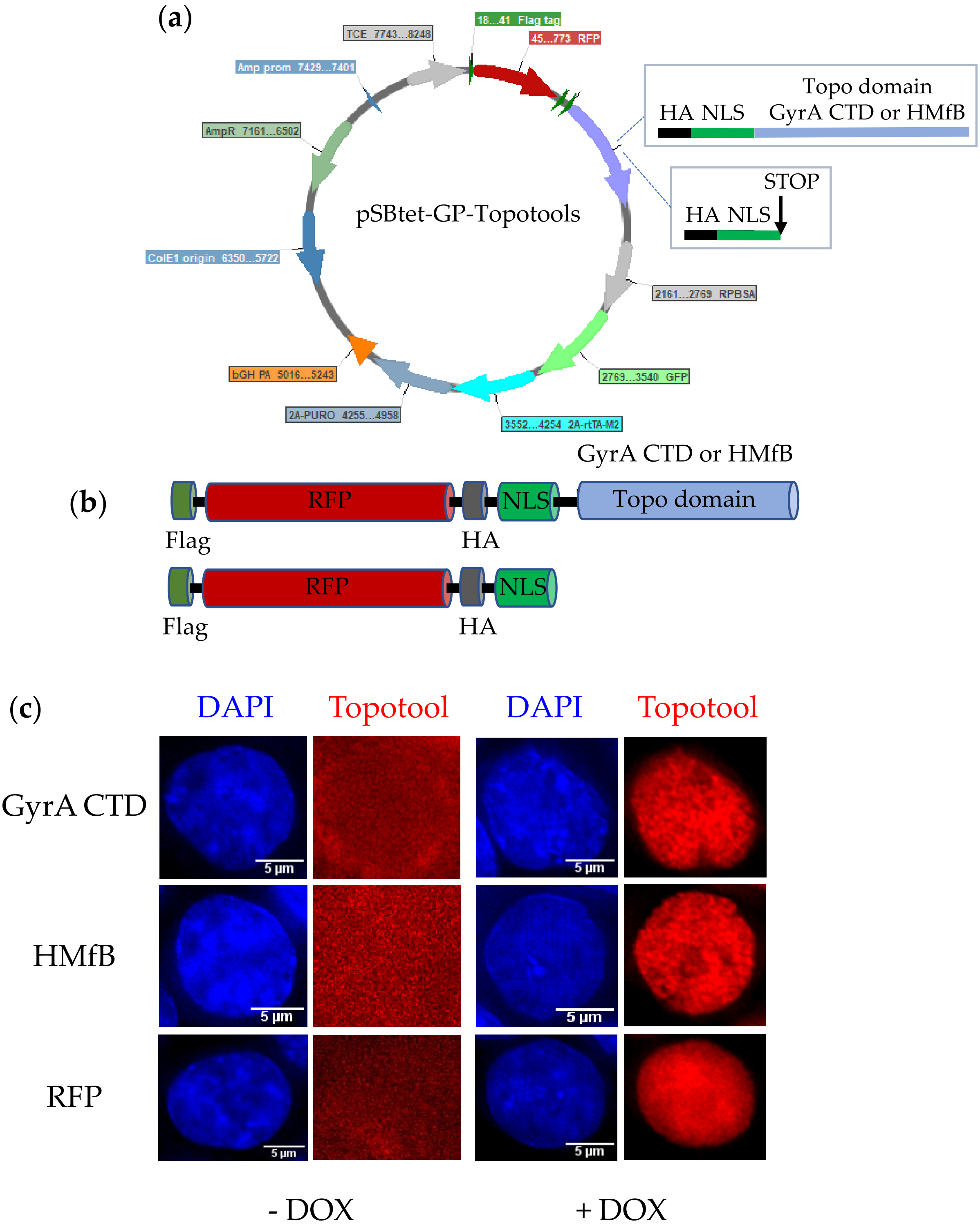

Figure S3. HCT116 Doxycyclin inducible system. (**a**) Vector used to generate a HCT116 cell line allowing Doxycyclin inducible expression of Topotools. (**b**) Schematics of the Topotools produced by this system. (**c**) Representative confocal images of the HCT116 cell lines constructed with the vectors in (**a**). Cells were fixed and no labelling or IF were performed.

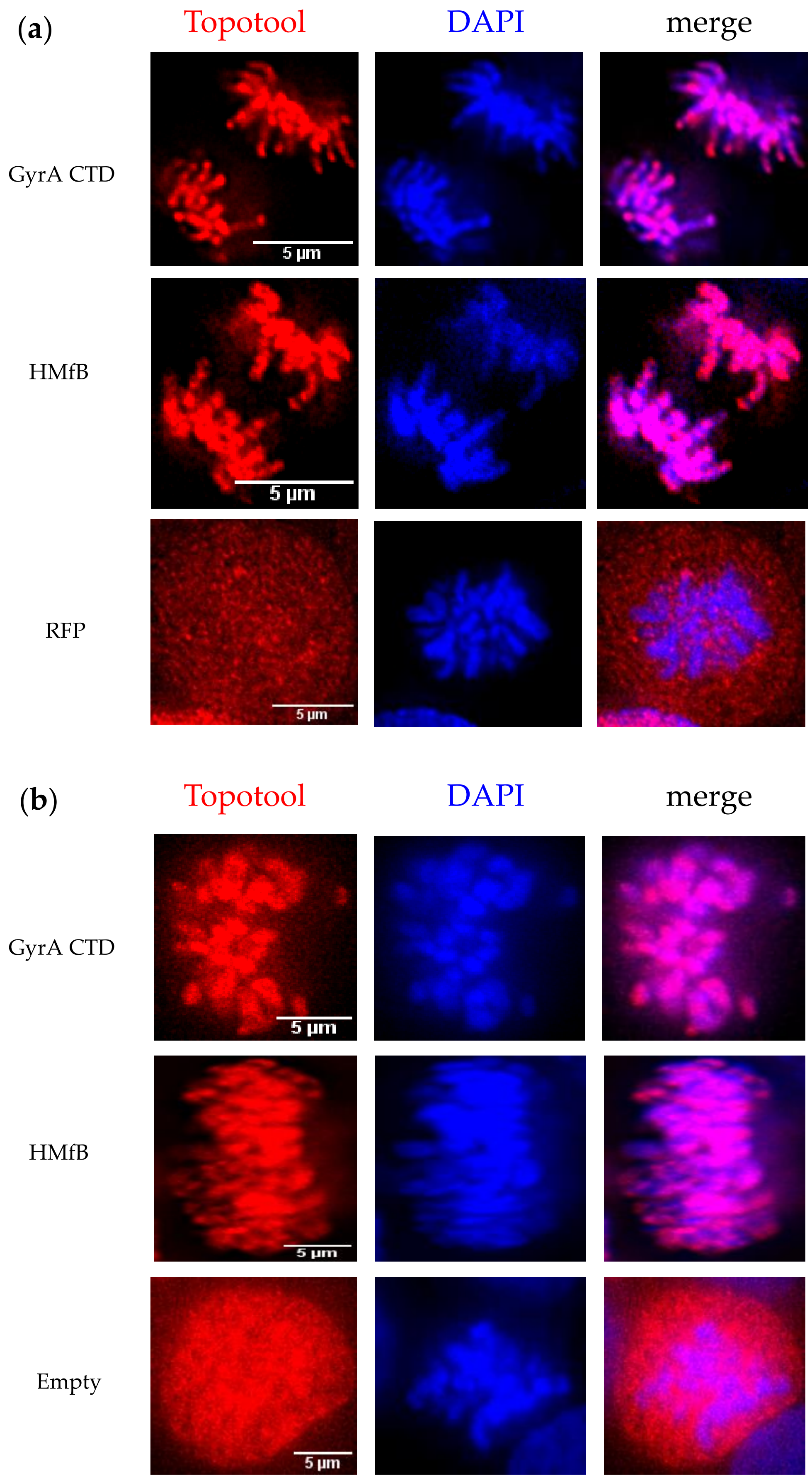

Figure S4. Topotools and chromatin during mitosis. Representative images of fixed HCT116 (**a**) and Hela-38 (**b**) cells expressing the Topotools or only RFP during mitosis.

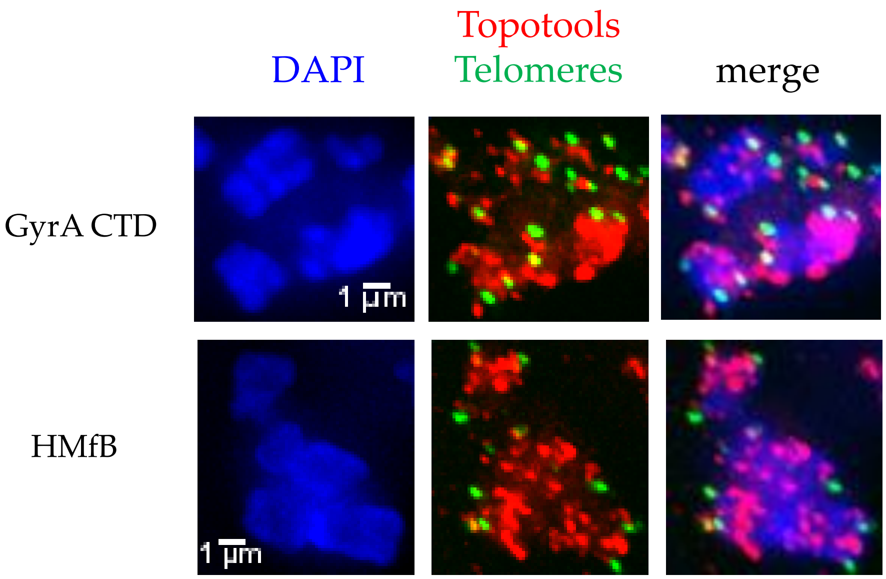

Figure S5. Partial telomeric localization of Topotools on metaphasic chromosomes. Representative images of metaphasic chromosomes spreads of HT1080 ST cells expressing the Topotools. Telomeres were visualized by PNA-FISH using a telomeric probe (green), Topotools by IF using the rat anti-HA antibody (red).

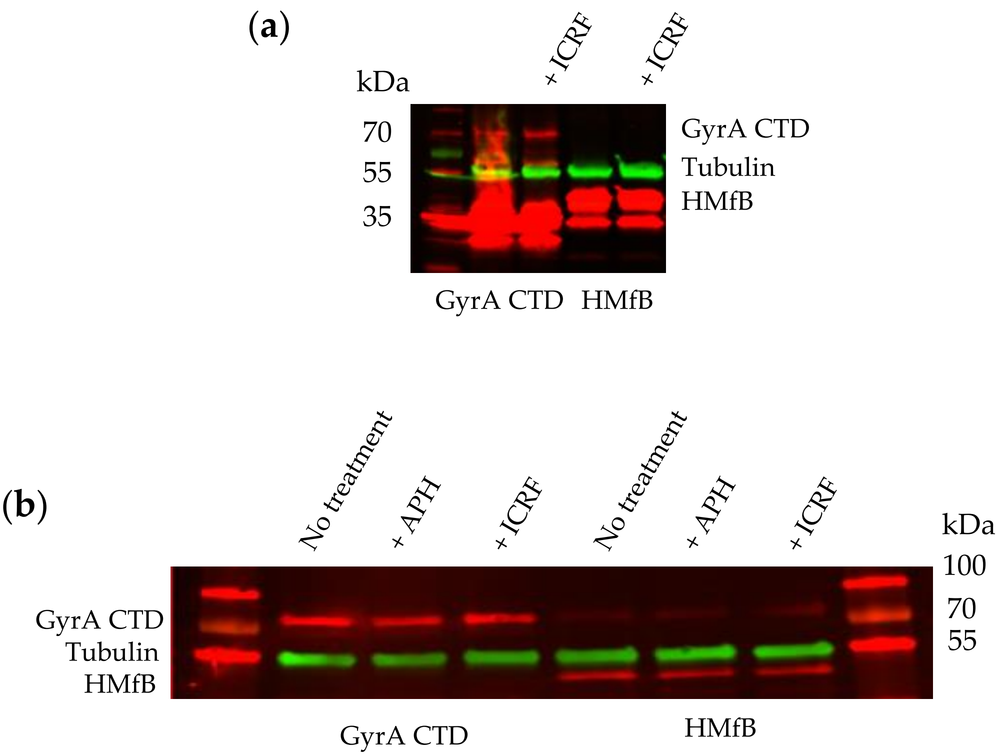

Figure S6. ICRF and APH do not cause alterations of Topotools expression. (**a**) Western blot showing Topotools and Tubulin expression in the experiment shown in Figure 3a. Anti-tRFP and anti-Tubulin antibodies were used. (**b**) Western blot showing Topotools and Tubulin expression in the ChIP experiments shown in Figure 3c. Rabbit anti-tRFP and mouse anti-Tubulin antibodies were used.

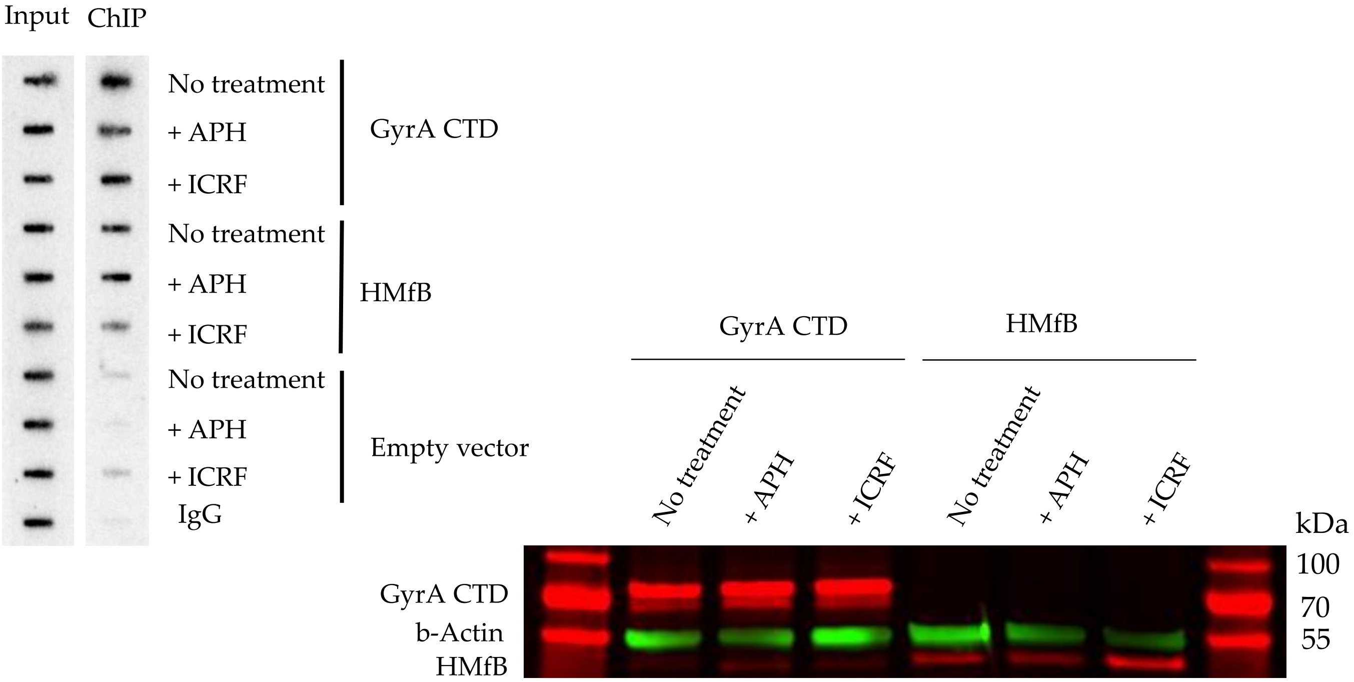

Figure S7. Topoisomerase 2 inhibition and replication stress cause a buildup of positive supercoils on telomeres. Biological replicate of the experiment shown in Figure 3c and d and associated western blot performed using Rabbit anti-tRFP and mouse anti-Tubulin antibodies.

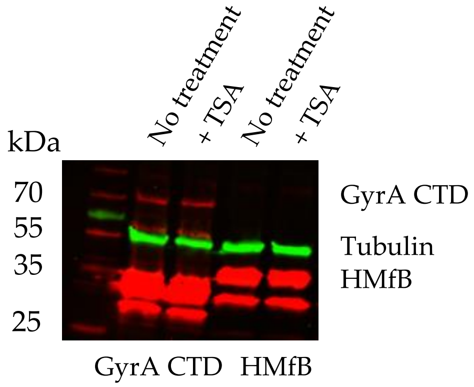

Figure S8. Trichostatin A (TSA) does not cause alterations of Topotools expression. Western blot showing Topotools and Tubulin expression in the experiment shown in Figure 4. Anti-tRFP and anti-Tubulin antibodies were used.

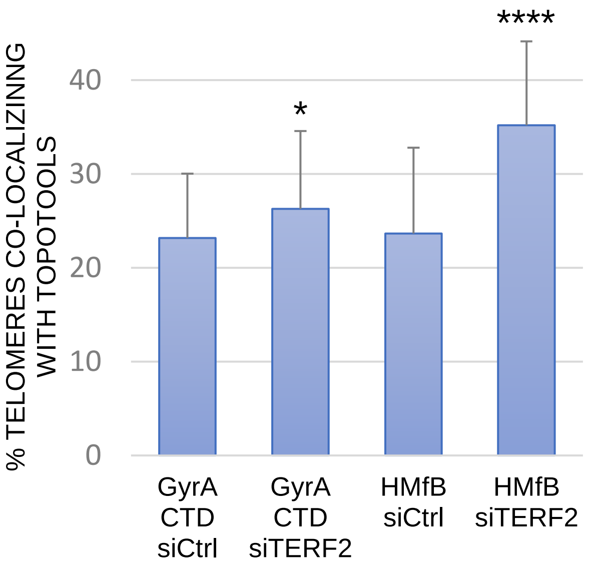

Figure S9. Knockdown of TRF2 causes supercoils build up on telomeres. Quantification of an independent experiment performed in the conditions used in Figure 5, but Topotools were detected using a rabbit anti-HA antibody. Statistics were performed compared to control using Kruskal-Wallis followed by Dunn tests. *$p<0.05$, **** $p<0.0001$.

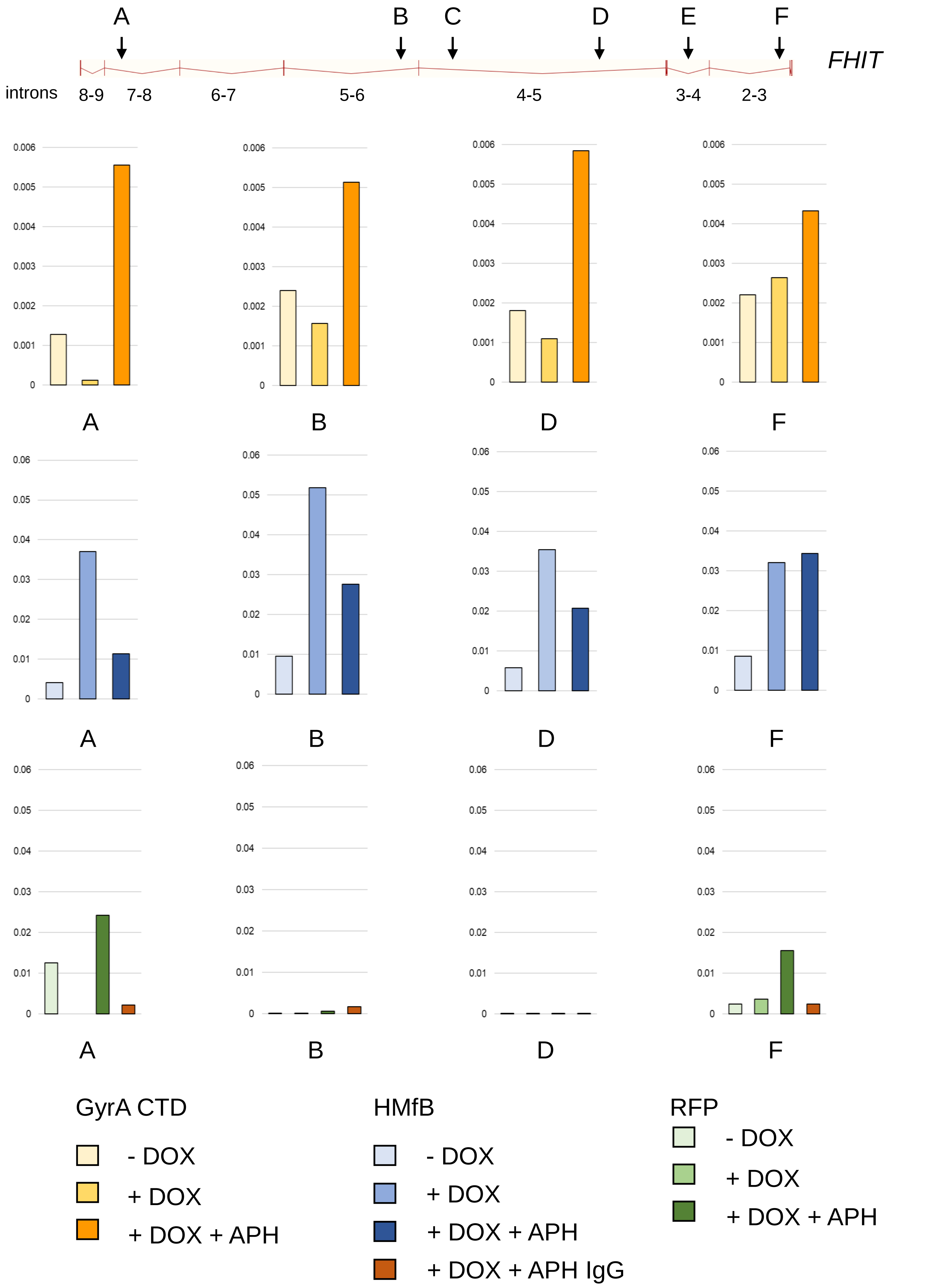

A
B
C
D
E
F
FHIT
introns
8-9
7-8
6-7
5-6
4-5
3-4
2-3
0.006
0.005
0.004
0.003
0.002
0.001
0
A
B
D
F
0.06
0.05
0.04
0.03
0.02
0.01
0
A
B
D
F
0.06
0.05
0.04
0.03
0.02
0.01
0
A
B
D
F
GyrA CTD
- DOX
+ DOX
+ DOX + APH
HMfB
- DOX
+ DOX
+ DOX + APH
+ DOX + APH IgG
RFP
- DOX
+ DOX
+ DOX + APH

Figure S10. Mild replication stress causes topological changes in the *FHIT* gene (biological replicate of the experiment shown in Figure 6). Top: graphical representation of the FHIT gene and position of primers used to analyze ChIP samples. Bottom: quantitative analysis of ChIP samples by qPCR using the primers above and performed on HCT116 cells expressing the Topotools and the RFP control. Three conditions were analyzed: DMSO (- DOX); Doxycyclin induction of Topotools expression (+ DOX); Doxycyclin induction and treatment with Aphidicolin (150 nM for 24 hours, + DOX + APH). Antibodies used were the rat anti-HA and a rat IgG.
